# In sickness and in health: A cross‐sectional analysis of concordance for musculoskeletal pain in 13,507 couples

**DOI:** 10.1002/ejp.744

**Published:** 2015-07-30

**Authors:** P. Campbell, M. Shraim, K.P. Jordan, K.M. Dunn

**Affiliations:** ^1^ Arthritis Research UK Primary Care CentreInstitute for Primary Care and Health SciencesKeele UniversityUK; ^2^ Department of Work EnvironmentUniversity of MassachusettsLowellUSA

## Abstract

**Background:**

Musculoskeletal pain conditions are common and create substantial burden for the individual and society. While research has shown concordance between couples for risk of some diseases, e.g. heart disease or diabetes, little information is available on such effects for musculoskeletal pain conditions. Our aims were to investigate the presence of concordance between couples for consultations about pain, and to examine theoretical influences on such concordance.

**Methods:**

This was a 1‐year cross‐sectional study of musculoskeletal pain consultations in a UK primary care database. In total 27,014 patients (13,507 couples) aged between 30 and 74 years were included. The main outcome measure was the presence of a musculoskeletal morbidity read code indicating a consultation for musculoskeletal conditions (any, back, neck, knee, shoulder, foot, osteoarthritis). Logistic regression was used to test associations with odds ratios (OR) and 95% confidence intervals (95% CI).

**Results:**

Patients whose partner had a musculoskeletal pain consultation were also more likely to consult for a musculoskeletal condition (OR 1.22, 95% CI 1.12–1.32). This association was found to be strongest for shoulder disorders (OR 1.91, 95% CI 1.06–3.47). No significant associations were found for other pain conditions.

**Conclusion:**

Results show that partner concordance is present for consultations for some musculoskeletal conditions but not others. Possible explanations for concordance include the shared health behaviours between couples leading to potential heightened awareness of symptoms. Given the high prevalence of musculoskeletal pain within populations, it may be worth considering further the mechanisms that explain partner concordance.

**What is already known about this topic?**

- Musculoskeletal conditions are common and create substantial burden for the individual and society.
- While research has shown concordance (i.e. shared characteristics) between couples for risk of some diseases, e.g. heart disease or diabetes, little information is available on such effects for musculoskeletal pain conditions.

**What does this study add?**

- This study has shown that partner concordance is present for musculoskeletal pain consultations within a primary care population.
- This study highlights patients' social context and supports consideration of the patient's household and family as a platform to better understand health outcomes.

## 1 Introduction

Research evidence demonstrates concordance between couples on the risk of illness and disease, most notably psychological well‐being (Stimpson et al., 2006; Kouros and Cummings, 2010), but also on diseases such as hypertension (Di Castelnuovo et al., 2009), diabetes (Khan et al., 2003) and heart disease (Schafer et al., 2004; Meyler et al., 2007). There are a number of suggested theoretical explanations for illness concordance between couples. One explanation is affective contagion, where it is suggested that emotional states are mutually shared between couples leading to concordance on beliefs and behaviours (Goodman and Shippy, 2002). Another explanation is shared environment and socialization, where couples share the same environmental factors such as housing, economic factors and social networks (Cardol et al., 2005; Meyler et al., 2007). There is also evidence of shared health behaviour within families, with a significant amount of engagement with health services explained at a family level (Cardol et al., 2007). One study, using medical record data (Hippisley‐Cox et al., 2002), considered a list of common illnesses (asthma, depression, diabetes, hypertension, ischaemic heart disease, hyperlipidaemia, stroke, peptic ulcer) and investigated whether having a partner with one of these illnesses increased the association of that illness in the other partner. They reported significant associations for asthma, depression, hypertension, hyperlipidaemia and peptic ulcers, offering a shared environment explanation. Importantly, the Hippisley‐Cox et al. paper did not find effects for other diseases such as diabetes, ischaemic heart disease or stroke, indicating the results found was not simply indicative of a general increased propensity to consult.

Little information exists on whether partner concordance exists for musculoskeletal pain conditions. Musculoskeletal pain represents a considerable burden worldwide: the recent global burden of disease findings showed that low back pain is the leading cause of years lived with disability (Vos et al., 2013). Burden is also reflected in healthcare consultations; musculoskeletal consultations account for around 20% of all consultations in UK primary care practices (McCormick et al., 1995; Jordan et al., 2007). Prevalence of persistent musculoskeletal pain is high, estimated at 25–32% (Wijnhoven et al., 2006), and recurrence rates are common (Ijzelenberg and Burdorf, 2004). Musculoskeletal pain conditions, therefore, have a major impact on the individual, healthcare and society (Bevan et al., Woolf and Pfleger, 2003).

We sought to investigate if there is an increased prevalence of primary care musculoskeletal consultations in those whose partners had also consulted for a musculoskeletal pain condition. The specific hypotheses of this study were: i) Does a musculoskeletal consultation in one partner increase the likelihood of a musculoskeletal consultation in the other partner? ii) Does having a musculoskeletal consultation in a specific body region or for a specific condition in one partner increase the likelihood of a musculoskeletal consultation in the same region or for the same condition in the other partner? iii) Do potential theoretical influences on concordance: affective contagion, shared deprivation and shared healthcare engagement explain the associations between partners' musculoskeletal pain consultations.

## 2 Methods

### 2.1 Setting

This was a 1‐year cross‐sectional study of medical consultations within primary care. Healthcare consultations were identified within the Consultations in Primary Care Archive (CiPCA), which is a validated database of the consultation records of 13 GP practices within North Staffordshire, UK (Porcheret et al., 2004; Jordan et al., 2007). CiPCA has been compared with other larger UK Primary Care National databases for musculoskeletal conditions, and has been shown to have comparable trends for age, sex and prevalence (Jordan et al., 2007). CiPCA also has ethics approval from the North Staffordshire Research Ethics Committee, and the quality of the database is assessed annually through training and feedback to practices (Jordan et al., 2007).

### 2.2 Participants and procedure

Couples were identified as two individuals both aged 30–74 years, having the same address, being of different gender, having a difference in age of no more than 15 years and having no other adult aged 30–74 within the household. These definitions follow similar inclusion criteria to Hippisley‐Cox et al. study (Hippisley‐Cox et al., 2002), and reduce the chance of including parent/adult child dyads. All included participants were registered at their respective GP practice for the period of analysis (31 December 2005–31 December 2006). Male partners were assigned as the exposure partner, with their corresponding female partner assigned as the outcome partner, similar to Hippisley‐Cox et al. Exposure was defined as a recorded read code for a musculoskeletal pain consultation in a male partner, with outcomes determined as recorded read code for a musculoskeletal pain consultation in the female partner during the same 12‐month period. A consultation was defined as a consultation at the practice, a home visit or by telephone that concluded with a recorded diagnostic code or symptom code. Multiple consultations on the same day were counted as ‘one’ contact.

### 2.3 Musculoskeletal pain consultations

We used the Read Code System to identify consultations for musculoskeletal pain conditions (NHS Information Authority, 2000). Read Codes are a common method for the computerized recording of morbidity in UK primary care (Benson, 2011). Following previous methodology (Jordan et al., 2010), all morbidity Read Codes relating to a musculoskeletal conditions within Read Code chapters N ‘Musculoskeletal and Connective Tissue Diseases’, R ‘Symptoms, Signs and Ill‐defined, S ‘Injury and Poisoning’ and 1 ‘History/Symptoms’ were used. All relevant codes were formed into the five most common consultation body regions (back, knee, neck, shoulder, foot), as well as codes for osteoarthritis consultations. A further category of ‘any musculoskeletal’ consultations were formed inclusive of the above body regions and conditions, as well as consultations for unspecified pain (e.g. arthralgia), widespread pain conditions and other single body regions where the proportion of consultations were too few to perform meaningful separate analysis (e.g. head, arm, elbow, wrist, hand, hip, pelvis, thigh and buttock).

### 2.4 Theoretical influences

To test for theoretical explanations of concordance, a number of proxy measures were employed from the data. Affective contagion influence was tested by identifying Read Codes during the 12‐month period relating to anxiety consultations (e.g. anxiety disorders, panic disorder) and mood state consultations (depressive disorders, dysthymia) in either partner, following previous methodology (Burton et al., 2013). We also extracted the number of times participants consulted in the study period (consultation frequency) to indicate shared healthcare engagement (i.e. healthcare use). Consultation frequency was dichotomized to indicate those within the top 20% of consultation frequency, per practice and per gender, following previous methodology (Foster et al., 2006). Home address postcodes were used to derive neighbourhood deprivation status for partners based on the UK Index of Multiple Deprivation 2007 to give indication of the shared deprivation (Office for National Statistics, [Link]). The deprivation variable was formed into three groups to indicate the 20% least deprived, 60% middle deprived and 20% most deprived following suggested methodology (Payne and Abel, 2012). Participant age was also recorded, and this was grouped into age bands (30–39 years, 40–49 years, 50–59 years, 60–69 years and 70 +  years) to account for the non‐linear relationship of musculoskeletal disorders and age (Thomas et al., 2007).

### 2.5 Statistical methods

Logistic regression was used to calculate odds ratios (OR) and 95% confidence intervals (95% CI) for the association of musculoskeletal pain consultations in female partners who have a male partner who has a musculoskeletal pain consultation, compared to female partners whose male partner has not consulted. Three stages of analysis were performed corresponding to the outlined study aims. Stage 1 considered the unadjusted associations for each type of musculoskeletal pain condition (any musculoskeletal, back, knee, neck, shoulder, foot and osteoarthritis disorders). Stage 2 reported on the independent influence of the theoretical explanations (affective contagion, shared healthcare engagement, shared deprivation and participant age) on the associations that were significant at stage 1. For example the presence/absence of a consultation for anxiety and/or mood state in the female partner, and in the male partner, was entered as covariates within the regression model to test for the influence of affective contagion on the association. Similarly, indication of being a frequent consulter (woman and partner) was entered as covariates to test for the influence of shared healthcare engagement, and deprivation status was entered to test for the influence of shared environmental factors. The regression model was adjusted only for female partner age bands due to the high level (>0.9) of correlation (collinearity) between partners' age. Multivariable adjustment was used in the third and final stage where all theoretical explanations, as outlined above (affective contagion, shared healthcare engagement, shared deprivation and female participant age) were included simultaneously. Further exploratory analysis was carried out to ascertain the proportions and odds ratios for the combined effects of affective contagion using logistic regression and 95% confidence intervals with adjustment for female participant age. For mood state, both singular effects of male and female partners, where one partner has anxiety/mood disorder but the other does not (mixed), and also where both partners have anxiety/mood disorder (both) were tested. Similarly for shared healthcare engagement analysis tested singular effects for male and female partners, where one partner is a frequent consulters and the other is not (mixed), and where both partners are frequent consulters (both) were tested. Analysis also considered shared area deprivation (both partners subject to the same influence).

## 3 Results

The total eligible population was 27,014 individuals, equating to 13,507 partner dyads. The mean age was 52 years, and the median number of consultations was 3 (within the 12‐month study period). In total, 12.3% (*n* = 3312) of the population did not have a recorded consultation within the 12‐month period. Exactly, 8292 (30.7%) patients were recorded as having a musculoskeletal pain consultation. Females had a slightly higher percentage of musculoskeletal pain consultation (32.4%) than males (29.0%). Table 1 outlines the characteristics of the cohort.

**Table 1:** Participant characteristics

	Mean	95% CI	Median	IQR
Age				
Male	53.0	52.8–53.2	53	44–62
Female	51.1	50.9–51.3	51	42–60
Consultation frequency over 12‐month period (All)	4.9	4.9–5.0	3	1–6
Males	4.2	4.2–4.3	3	1–6
Females	5.6	5.5–5.7	4	2–8

Musculoskeletal consultation prevalence		
Males	Females	Both partners
3917 (29.0%)	4375 (32.4%)	1457 (10.78%)

Musculoskeletal pain consultations		
	Male	Female
	Number (%)	Number (%)
Back	804 (6.0%)	852 (6.3%)
Knee	521 (3.9%)	497 (3.7%)
Neck	269 (2.0%)	360 (2.7%)
Shoulder	301 (2.2%)	267 (2.0%)
Foot	237 (1.8%)	272 (2.0%)
Osteoarthritis	311 (2.3%)	350 (2.6%)

IQR, interquartile range; CI, confidence interval.

Unadjusted logistic regression results (Table 2) show that females whose partner had consulted for any musculoskeletal pain condition had a significantly increased likelihood of a musculoskeletal pain consultation when compared to those whose partner had no recorded musculoskeletal pain consultation (OR 1.35, 95% CI 1.25–1.46). Similarly, the odds of consulting for osteoarthritis were more than doubled for females whose male partner had also consulted about osteoarthritis (OR 2.38, 95% CI 1.46–3.88). Musculoskeletal shoulder consultations were also more likely if the male partner had such a consultation (OR 2.11, 95% CI 1.17–3.81). Other regional musculoskeletal pain consultations (back, knee, neck, foot) showed no significant associations.

**Table 2:** Unadjusted associations of concordance for MSK pain in couples

Disorder	Consultation percentages		Odds ratio	95% confidence intervals
	Female (male partner without consultation) (%)	Female (male partner with consultation) (%)		
Any MSK	26.9	33.3	1.35	1.25–1.46^b^
Back	6.0	5.9	0.98	0.73–1.32
Knee	3.9	3.8	0.99	0.62–1.58
Neck	2.0	1.4	0.69	0.28–1.68
Shoulder	2.2	4.5	2.11	1.17–3.81^a^
Foot	1.8	1.1	0.62	0.20–1.95
Osteoarthritis	2.2	5.1	2.38	1.46–3.88^b^

MSK – Musculoskeletal.

a *P *<* *0.05 (two‐sided).

b *P *<* *0.001 (two‐sided).

Adjusted results (Table 3) for any musculoskeletal pain consultation show that there was no marked effect on the strength of association of a musculoskeletal pain consultation for the female partner when adjusted for the influence of affective contagion, shared deprivation or age. Only adjusting for shared healthcare engagement (consultation frequency) led to a small but noticeable reduction in association of consulting, (OR reduced from 1.35 to 1.22). Results for osteoarthritis consultations showed no effect from adjustment for affective contagion or shared deprivation, but there was a reduction in odds following adjustment for shared healthcare engagement (OR 2.38–1.86). However, age had the largest impact on the strength of association on concordant osteoarthritis consultation, with a marked reduction in odds (OR 2.38–1.37) leading to a non‐significant association. Results for adjustment for concordance in shoulder consultations show no perceptible effect from affective contagion, shared deprivation or age, but a small reduction in strength of association (OR 2.11–1.92) for shared healthcare engagement. The final multivariable model, with simultaneous adjustment for affective contagion, shared healthcare engagement, shared deprivation and participant age showed increased strength of association for a consultation for any musculoskeletal pain condition for females if their partner also consulted for a musculoskeletal pain consultation (OR 1.20; 95% CI 1.10, 1.31). The partner association for shoulder conditions was still significant after multivariable adjustment (OR 1.91; 95% CI 1.05, 3.46), but the association for osteoarthritis consultations was non‐significant.

**Table 3:** Multivariable models of couple concordance for musculoskeletal pain consultations

		Adjusted models				
Pain condition	Unadjusted	Affective contagion	Shared healthcare engagement	Shared deprivation	Participant age (females)	Final multivariable model^c^
Any MSK	1.35 (1.25–1.46)^b^	1.34 (1.24–1.45)^b^	1.22 (1.13–1.33)^b^	1.35 (1.25–1.46)^b^	1.31 (1.21–1.42)^b^	1.20 (1.10–1.31)^b^
Osteoarthritis disorders	2.38 (1.46–3.88)^b^	2.40 (1.47–3.91)^b^	1.86 (1.13–3.06)^a^	2.37 (1.45–3.86)^b^	1.37 (0.83–2.24)	1.30 (0.79–2.14)
Shoulder	2.11 (1.17–3.81)^a^	2.10 (1.16–3.80)^a^	1.92 (1.06–3.47)^a^	2.11 (1.17–3.80)^a^	2.05 (1.14–3.71)^a^	1.91 (1.05–3.46)^a^

Values are given as OR (95% CI). MSK, musculoskeletal; OR, odds ratio; CI, confidence interval.

a *P *<* *0.05 (two‐sided).

b *P ≤ *0.001 (two‐sided).

c Adjusted for affective contagion, shared healthcare engagement, shared deprivation and female age.

Results of the analysis for any musculoskeletal consultations (Table 4) show both female and male partner mood state, and anxiety state, independently associate with any musculoskeletal consultation. This effect is stronger when both partners are coded for a mood disorder, with a non‐significant increasing trend when both partners are coded for anxiety. Similarly, consultation frequency has a strong association with any musculoskeletal consultation if the females or males are frequent consulters, with the strongest effect when both partners are frequent consulters. Shared deprivation was also shown to increase the associated odds for any musculoskeletal consultation with a 30% increase for those within the high deprivation group compared to those in the lowest deprivation group. Results on age show there was a gradual increase in the prevalence of any musculoskeletal pain consultations with increasing age.

**Table 4:** Influence of affective contagion, shared health behaviours, shared deprivation and age on female musculoskeletal pain consultation

	Influence	Influence present	Percentage females with any musculoskeletal pain consultation	OR (95% CI)	OR (95% CI) adjusted for female age
Affective contagion	Female anxiety	No	31.9	1.50 (1.28, 1.75)	1.52 (1.30, 1.78)
		Yes	41.2		
	Male anxiety	No	32.2	1.29 (1.03, 1.62)	1.32 (1.05, 1.65)
		Yes	38.1		
	No anxiety (both)	Yes	31.8	Reference	Reference
	Mixed anxiety^a^	Yes	39.9	1.43 (1.24, 1.63)	1.44 (1.26, 1.66)
	Both anxiety^b^	Yes	43.2	1.63 (0.90, 2.96)	1.70 (0.93, 3.10)
	Female mood	No	32.1	1.58 (1.29, 1.93)	1.69 (1.38, 2.08)
		Yes	42.7		
	Male mood	No	32.3	1.38 (1.02, 1.86)	1.41 (1.05, 1.90)
		Yes	39.7		
	No mood (both)	Yes	32.0	Reference	Reference
	Mixed mood^a^	Yes	40.9	1.47 (1.23, 1.75)	1.55 (1.30, 1.85)
	Both mood^b^	Yes	52.4	2.34 (0.99, 5.51)	2.56 (1.08, 6.07)
Shared healthcare engagement	Female frequent consulter	No	25.5	3.97 (3.65, 4.33)	3.89 (3.57, 4.24)
		Yes	57.6		
	Male frequent consulter	No	30.2	1.54 (1.42, 1.68)	1.38 (1.27, 1.51)
		Yes	40.1		
	Both not frequent^b^	Yes	24.4	Reference	Reference
	Mixed frequent^a^	Yes	42.2	2.26 (2.09, 2.45)	2.18 (2.01, 2.36)
	Both frequent^b^	Yes	63.4	5.38 (4.65, 6.21)	4.95 (4.28, 5.73)
Shared deprivation	Low deprivation	Yes	31.6	Reference	Reference
	Middle deprivation	Yes	31.3	0.99 (0.90, 1.09)	1.02 (0.93, 1.12)
	High deprivation	Yes	36.5	1.25 (1.12, 1.40)	1.30 (1.16, 1.46)
Age bands of women	30–39		25.0	Reference	
	40–49		28.9	1.22 (1.09, 1.37)	
	50–59		36.0	1.69 (1.51, 1.89)	
	60–69		36.7	1.74 (1.55, 1.95)	
	70+		41.3	2.11 (1.76, 2.53)	

OR, odds ratio; CI, confidence interval.

a Mixed = where one partner has potential influence and other does not.

b Both = where both partners have potential influence.

## 4 Discussion

Female partners were more likely to have a consultation for a musculoskeletal pain condition if their male partner had also consulted for a musculoskeletal pain condition even after adjustment for potential theoretical influences in both partners (affective contagion, shared healthcare engagement, shared deprivation and age). This association was strongest for shoulder problems. These findings highlight potential social effects on the rates of musculoskeletal pain consultations within primary care.

Although, to our knowledge, there are no directly comparable musculoskeletal partner risk studies using medical records, this current study does show similarities in methodology to the Hippisley‐Cox et al. study, on partner risk for other common diseases using medical record data (Hippisley‐Cox et al., 2002). In addition, there is a key advantage to this current study as we considered and accounted for the consultation frequency of our participants (over a 12‐month period), therefore lessening the chance of any associations being explained by virtue of consultation frequency alone. There are a number of theoretical influences that might explain illness concordance between couples. One notable influence from the literature is affective contagion that couples will be influenced by sharing similar mood states (Goodman and Shippy, 2002). We attempted to assess this effect by adjusting for participants who were coded as having a mood state disorder or an anxiety disorder. Results showed little change in any of the significant musculoskeletal associations within the logistic regression model when adjusting for these effects, and so musculoskeletal consultation influence from partners may not be significantly driven by mood state, in the self or in the partner. However examination of the direct influence (Table 4) did show increases in any musculoskeletal pain consultations based on whether either the female or male partner, or more so if both, had a mood state or anxiety disorder, indicating some effect is present. Closely related to the affective contagion hypothesis is the shared environment and socialization hypothesis (Meyler et al., 2007). This is where couples share the same environment, resources and behaviour, with good evidence that couples share similar lifestyles as a result of their shared environment (Jurj et al., 2006). We attempted to assess this hypothesis by adjusting for a measure of neighbourhood deprivation, and the results show no effect of deprivation on the partner concordance associations within the regression model, although a slight increase in odds was shown within the direct model for any musculoskeletal consultations. However, we did not include measures of socialization or lifestyle (e.g. diet, smoking status, alcohol intake, obesity, physical fitness, social support and network, family income) that are indicative of deprivation status, as these factors are less likely to be recorded by GPs, and it may be these influences are more likely to lead to greater illness concordance (Ferrer et al., 2005). Adjustment for shared healthcare engagement, using participant consultation frequency, did show an effect on all significant associations of musculoskeletal concordance (any musculoskeletal, shoulder and osteoarthritis consultations). This suggests that some of the explanation for concordance might be explained by shared health beliefs and health behaviours of couples (i.e. motivational factors to consult). Cardol et al. has demonstrated family influence on why someone decides to seek healthcare (Cardol et al., 2006, 2007). It may be that when a partner consults for a musculoskeletal pain condition, this initiates a consultation by the other partner because of a heightened awareness of the symptoms, a shared belief on what the illness is, and also the benefits to be had from treatment. Concordance between partners in osteoarthritis consultations were shown to be influenced by adjustment for participant age, with age explaining most of the association found. This finding fits with evidence that age is a significant risk factor for osteoarthritis conditions, and this was the single shared factor that explained the concordance effect (Felson et al., 2000; Thomas et al., 2007). However, the results show partner concordance in shoulder consultations was still significant after adjustment for all influences. One possible explanation could lie with shared behaviour between couples, e.g. shared hobbies or activities that place the shoulder at increased risk. Another reason may be comorbidity with other diseases, which may have increased the chance of concordance (unfortunately not assessed within this current study). There is good evidence that shoulder problems, such as frozen shoulder, are linked to, and have higher prevalence in, other conditions such as diabetes, hypothyroidism and hypoadrenalism (Dias et al., 2005; Milgrom et al., 2008). There is also evidence that shoulder problems are linked to cardiac, pulmonary and stroke conditions (Kuijpers et al., 2004). All of these conditions have been shown to be influenced by partner concordance (Khan et al., 2003; Meyler et al., 2007; Di Castelnuovo et al., 2009), and it may be that these conditions are shared by couples leading to a stronger concordance effect for shoulder consultations. Finally, the results show some concordance when all musculoskeletal pain consultation codes were considered (any musculoskeletal). It may be that the inclusion of shoulder consultations and osteoarthritis consultations within this category led to this effect. However, examination of the independent contributions of affective contagion, healthcare engagement, shared deprivation and age (Table 4) do demonstrate direct associations with any musculoskeletal pain consultations, and further research is needed to understand the mechanisms as to why such influence is present. The choice of female musculoskeletal consultation as the outcome in this current study was arbitrary and followed the Hippisley‐Cox et al. (2002) design. However, we did perform a replication of the analysis shown in Table 4 to consider whether there was any difference when male musculoskeletal consultation was the outcome (see Supporting Information Table S1). The results of this additional analysis show broadly similar effects and trends; however, proportions overall are reduced because musculoskeletal consultation proportion frequency is less in males, and the regression effects (odds ratios) suggest that males appear less influenced by affective contagion and shared healthcare engagement compared to females.

### 4.1 Strengths and weaknesses of this study

A major strength of this study is the large sample size, representative of a general population sample of couples aged between 30 and 74, given that over 97% of the UK population are registered with a primary care GP (Bowling, 1997). The study also considered the effects of consultation frequency which was a notable weakness in previous studies. Use of consultation data also reduces the risk of recall and selection bias, shown to influence questionnaire‐based designs (Podsakoff et al., 2003). Furthermore, the CiPCA database has been shown to give comparable musculoskeletal prevalence figures to UK National Primary Care databases (Jordan et al., 2007), and such medical record databases have been shown to be suitable for epidemiological studies (Hassey et al., 2001; Benson, 2011). This study has demonstrated musculoskeletal pain consultation concordance between couples, as well as tested theoretical influences on concordance, which have shown some effect is present. However, the measures we used to represent theoretical influences are limited. The CiPCA data set, as with all primary care databases, is restricted on information about shared lifestyles (e.g. diet, exercise, alcohol intake, smoking status), health behaviours and health beliefs between couples, as information such as this are not routinely recorded by GPs (e.g. diet, exercise, health beliefs), or are less well recorded compared to the index condition for which the patient has visited their GP. For example, some recent epidemiological studies, (Mulnier et al., 2006; Delaney et al., 2007; Osborn et al., 2007) investigating risk factors for various illnesses (cardiovascular, diabetes, metal health), report significant levels of missing data for information on BMI (obesity), alcohol intake and smoking behaviour within UK primary care databases, and research is now calling for greater psychosocial information, elicited from patients, to be added to electronic health records to address this issue (Glasgow et al., 2012).

In addition, while we did include the area level of deprivation for the household, we do not have any specific information relating to actual financial status of each partner, or the specifics of deprivation within each household; information such as this may have been more sensitive within our analysis. Furthermore, due to the cross‐sectional design, we have no information on which partner consulted for their musculoskeletal pain condition first (incident exposure), no information on the duration of each musculoskeletal pain condition (e.g. a consultation does not signify the start of an episode), no information on relationship length between couples prior to taking part and so no way of testing the development of concordance between couples. We also have no information on couples who are aged below 30 years or same sex couples where differences may have been present. Prospective designs are now needed to establish both causality and how concordance develops. It is also true that not everybody who has a musculoskeletal pain condition will consult, and there may well be partner influence on not consulting that we were unable to test within this data set.

### 4.2 Clinical relevance

In terms of clinical relevance, from an individual patient perspective, the reported odds ratios are relatively small and we would not advocate intervention at the partner level (i.e. treating the partner) to reduce such effects in presenting patients. However, bearing in mind the high percentage of the population who consult about musculoskeletal pain conditions (Jordan et al., 2010), the results may be more meaningful from a public health standpoint. Research has already shown that interventions targeting modifiable lifestyle factors at a partner and family level can reduce the impact of conditions such as diabetes and coronary heart disease (Pyke et al., 1996; Khan et al., 2003), and that a significant amount of variance in individual health outcomes can be explained at a family level (Ferrer et al., 2005). There may be the potential to consider family level interventions for musculoskeletal conditions. For example, Martire et al., 2004 demonstrate, in a review and meta‐analysis of family level interventions, that positive effects on outcomes (e.g. index condition for patient, psychological outcomes for patient and family member) are present in both the patient and family member if a family member is involved in the treatment process. However, this current study's findings are too limited to give indication on whether concordance can be beneficial to the patient (i.e. a person is influenced to get appropriate and timely treatment based on their partners' experience) or not (i.e. shared maladaptive behaviours between partners that function as barriers to recovery). Further work is now needed to consider the impact of concordance on outcomes for those with musculoskeletal pain.

## 5 Conclusion

This study has demonstrated an increase in the likelihood of a musculoskeletal pain consultation if a partner also consults for a musculoskeletal pain condition. Possible explanations include shared deprivation and shared healthcare engagement that couples may have. This study highlights patients' social context and supports consideration of the patient's household and family as a platform to better understand health outcomes.

## Supporting information


**Table S1.** Influence of affective contagion, shared health behaviours, shared deprivation and age on male musculoskeletal pain consultation.
