# Supplementary material for: In sickness and in health: A cross‐sectional analysis of concordance for musculoskeletal pain in 13,507 couples
Source: Eur J Pain. 2015 Jul 30;20(3):438–46. doi: 10.1002/ejp.744 (PMC4762537; doi:10.1002/ejp.744)
Supplement: Supplementary file 1 — Table S1. Influence of affective contagion, shared health behaviours, shared deprivation and age on male musculoskeletal pain consultation. [file EJP-20-438-s001.docx]

**Supplementary Table 1**

Influence of affective contagion, shared health behaviours, shared deprivation and age on male musculoskeletal pain consultation

| Influence | | Influence present | Percentage males with any musculoskeletal pain consultation | OR (95% CI) | OR (95% CI) adjusted for male age |
| --- | --- | --- | --- | --- | --- |
| Affective contagion | Male anxiety | No | 28.7% | 1.80 (1.44, 2.20) | 1.83 (1.47, 2.28) |
|  |  | Yes | 42.0% |  |  |
|  | Female anxiety | No | 28.8% | 1.24 (1.05, 1.46) | 1.25 (1.06, 1.47) |
|  |  | Yes | 33.3% |  |  |
|  | No anxiety (both) | Yes | 28.4% | Reference | Reference |
|  | *Mixed anxiety | Yes | 36.1% | 1.42 (1.24, 1.64) | 1.44 (1.25, 1.65) |
|  | ^#^Both anxiety | Yes | 36.4% | 1.44 (0.78, 2.66) | 1.48 (0.80, 2.75) |
|  | Male mood | No | 28.9% | 1.48 (1.09, 2.00) | 1.50 (1.11, 2.03) |
|  |  | Yes | 37.5% |  |  |
|  | Female mood | No | 28.9% | 1.12 (0.90, 1.39) | 1.02 (1.01, 1.02) |
|  |  | Yes | 31.3% |  |  |
|  | No mood (both) | Yes | 28.8% | Reference | Reference |
|  | *Mixed mood | Yes | 31.8% | 1.15 (0.96, 1.38) | 1.19 (0.99, 1.44) |
|  | ^#^Both mood | Yes | 52.4% | 2.71 (1.15, 6.39) | 2.88 (1.22, 6.81) |
|  |  |  |  |  |  |
| Shared  healthcare engagement | Male frequent consulter | No | 23.5% | 3.65 (3.34, 4.00) | 3.55 (3.23, 3.90) |
|  |  | Yes | 52.9% |  |  |
|  | Female frequent consulter | No | 27.8% | 1.34 (1.22, 1.47) | 1.28 (1.17, 1.41) |
|  |  | Yes | 34.0% |  |  |
|  | ^#^Both not frequent | Yes | 22.1% | Reference | Reference |
|  | *Mixed frequent | Yes | 37.7% | 2.13 (1.96, 2.31) | 2.07 (1.91, 2.25) |
|  | ^#^Both frequent | Yes | 54.9% | 4.29 (3.72, 4.94) | 4.02 (3.48, 4.64) |
|  |  |  |  |  |  |
| Shared deprivation | Low deprivation | Yes | 27.5% | Reference | Reference |
|  | Middle deprivation | Yes | 28.9% | 1.07 (0.97, 1.18) | 1.09 (0.99, 1.21) |
|  | High deprivation | Yes | 30.9% | 1.18 (1.05, 1.32) | 1.21 (1.08, 1.36) |
|  |  |  |  |  |  |
| Age bands of males | 30 to 39 |  | 23.1% | Reference |  |
|  | 40 to 49 |  | 25.7% | 1.15 (1.01, 1.31) |  |
|  | 50 to 59 |  | 30.2% | 1.44 (1.27, 1.63) |  |
|  | 60 to 69 |  | 32.7% | 1.61 (1.42, 1.83) |  |
|  | 70+ |  | 35.3% | 1.82 (1.55, 2.13) |  |
| OR – Odds Ratio, CI – Confidence interval, * Mixed = where one partner has potential influence and other does not, ^#^Both = where both partners have potential influence. | | | | | |
